# Can gentamicin-containing collagen sponge be used for the protection against leakage following low anterior resection with total mesorectal excision?

**DOI:** 10.1007/s10151-014-1139-7

**Published:** 2014-04-03

**Authors:** B. Szynglarewicz, M. Ekiert, J. Forgacz, R. Matkowski

**Affiliations:** 1 Department of Surgical Oncology, Lower Silesian Oncology Center - Regional Comprehensive Cancer Center, pl. Hirszfelda 12, 53-413 Wrocław, Poland; 2 Department of Oncology and Division of Clinical Oncology, Wroclaw Medical University, ul. Borowska 213, 50-556 Wrocław, Poland; 3 Department of Oncology and Division of Surgical Oncology, Wroclaw Medical University, pl. Hirszfelda 12, 53-413 Wrocław, Poland

Dear Sir,

Anterior resection with total mesorectal excision (TME) may result in an increased risk of the anastomotic leakage (AL) because of the short rectal remnant and local oxygen deficiency in the anastomosis associated with the reduced distal blood supply. Moreover, it produces a large splinted cavity within the pelvis, conducive to exudate retention and formation of a hematoma which may become infected. The presence of AL impairs both late functional and oncological outcomes [1]. On the other hand, recent technological innovations such as resorbable implants offer new possibilities to protect the anastomosis and reduce the consequences of leakage. Some years ago, we reported our initial results of the wrapping of anastomosis with the gentamicin-collagen sponge (GCS) as a potential preventive maneuver against the AL—probably limiting the leakage intensity and reducing its clinical symptoms [2].

Now we would like to share our single-center experience of 158 consecutive patients with T1–T3 low rectal cancer who underwent anterior resection with TME and straight end-to-end anastomosis with double-stapling technique. Sixty-five (41 %) patients with T3 or N+ tumors received preoperative radiotherapy 5 × 5 Gy. All anastomoses were wrapped with GCS 10 × 10 × 0.5 cm containing 130 mg of gentamicin sulfate and 280 mg purified bovine tendon type I collagen, which was placed deep in the pre-sacral area at the level of the levators. The sponge was formed and pressed to the bowel wall. A special effort was made to ensure that it’s location and stability were satisfactory (Fig. 1). No patient in this series had a diverting stoma.

**Fig. 1:**
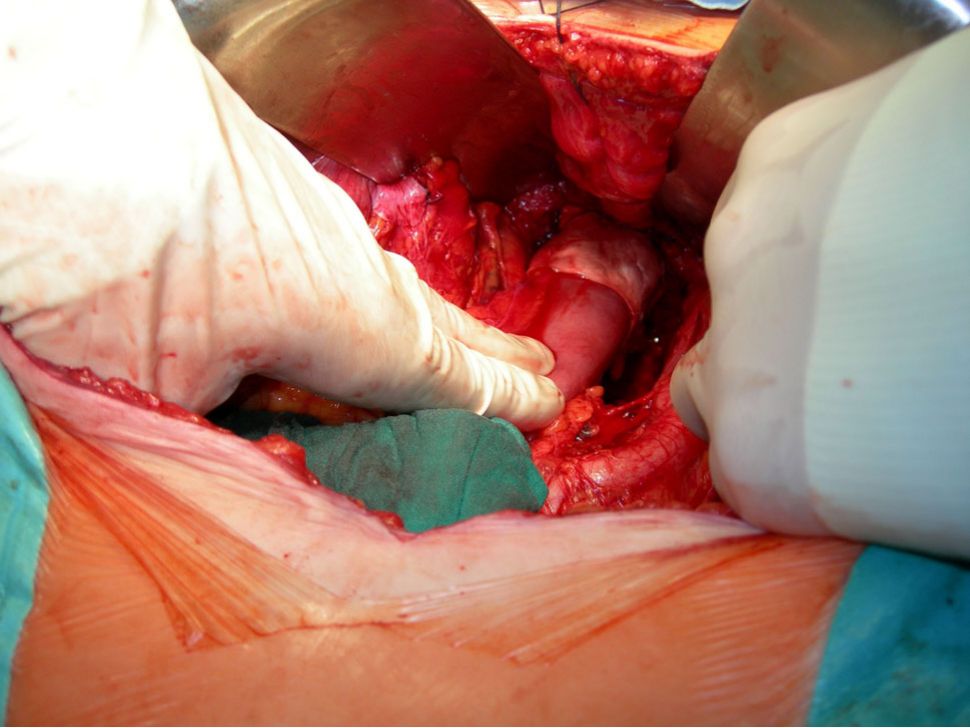
Low anastomosis wrapped with GCS

Gentamicin-collagen sponge was applied without any technical difficulties and was well tolerated. Neither sponge-related adverse reactions nor drain blockage were noticed. AL developed in five patients (3.2 %) and was associated with peritonitis in one patient (1.6 %), pelvic abscess in another (both without protective stoma) and gas or feculent discharge from the pelvic drain in three others (with stoma). The median time to AL diagnosis was 8 days (range 3–15 days) following surgery. Patients with peritonitis and abscess underwent surgical reintervention: peritoneal lavage and defunctioning transversostomy. The remaining patients had only minor AL and were effectively treated with pelvic lavage through the drain, total parenteral nutrition and antibiotic therapy. There was no leakage-related mortality.

AL incidence in our group seems to be relatively low when compared to the vast majority of other series (8–23 %) [1]. Only a few papers concerning the use of GCS in colorectal surgery have been published so far. It remains a subject of debate: findings are contradictory, the importance of results is limited and no statistically significant conclusions can be drawn [3]. Also, little is known about the impact of GCS on anastomosis healing. Some studies suggest local gentamicin has positive effects on collagen content and metabolism. Quicker mucosal, muscular and extra-cellular matrix repair was observed in an experimental study in dogs [4]. Other investigators reported that intra-abdominal application of gentamicin can enhance the healing of anastomosis and increase the collagen type I/III in rats [5]. This topic warrants further investigation. Our recent results may suggest that the low incidence of symptomatic AL might be at least partially influenced by GCS application which could secure the anastomosis area—it reduces tissue exudation and fluids accumulation at the pelvis cavity and also has a local antibacterial and hemostatic activity. Potential benefit from GCS may also be associated with the ability to reduce the extent of dehiscence and its severe consequences and limit pelvic abscess formation, peritonitis and septicemia without the impact on subclinical failure. This may explain the favorable clinical course of AL in our series.

Investigation with a longer follow-up are needed to evaluate the pattern and incidence of possible late consequences of GCS implantation (e.g., anastomotic stricture) as well as its cost-effectiveness.

## References

[1] Taflampas P, Christodoulakis M, Tsiftsis DD (2009). Anastomotic leakage after low anterior resection for rectal cancer: facts, obscurity, and fiction. Surg Today.

[2] Szynglarewicz B, Matkowski R, Gisterek I (2007). Implantation of gentamicin-containing collagen sponge following anterior resection for rectal carcinoma: can it decrease the leakage risk?. Colorectal Dis.

[3] de Bruin AF, Gosselink MP, van der Harst E, Rutten HJ (2010). Local application of gentamicin collagen implants in the prophylaxis of surgical site infections following gastrointestinal surgery: a review of clinical experience. Tech Coloproctol.

[4] Mutter D, Aprahamian M, Tiollier J, Sonzini P, Marescaux J (1997). Evaluation of human collagen biomaterials in the healing of colonic anastomoses in dogs. Eur J Surg.

[5] Binnebosel M, Junge K, Kaemmer DA (2009). Intraperitoneally applied gentamicin increases collagen content and mechanical stability of colon anastomosis in rats. Int J Colorectal Dis.

